# Hope for GWAS: Relevant Risk Genes Uncovered from GWAS Statistical Noise

**DOI:** 10.3390/ijms151017601

**Published:** 2014-09-29

**Authors:** Catarina Correia, Yoan Diekmann, Astrid M. Vicente, José B. Pereira-Leal, Emil Alexov

**Affiliations:** 1 Instituto Nacional de Saúde Doutor Ricardo Jorge, Av. Padre Cruz, Lisboa 1649-016, Portugal; E-Mail: astrid.vicente@insa.min-saude.pt; 2 Centre for Biodiversity, Functional & Integrative Genomics, Faculty of Sciences, University of Lisboa, Lisboa 1749-016, Portugal; 3 Instituto Gulbenkian de Ciência, Oeiras 2780-156, Portugal; E-Mails: ydiekmann@igc.gulbenkian.pt (Y.D.); jleal@igc.gulbenkian.pt (J.B.P.-L.)

**Keywords:** genome-wide association studies (GWAS), missing heritability, protein-protein interaction networks, functional coherence

## Abstract

Hundreds of genetic variants have been associated to common diseases through genome-wide association studies (GWAS), yet there are limits to current approaches in detecting true small effect risk variants against a background of false positive findings. Here we addressed the missing heritability problem, aiming to test whether there are indeed risk variants within GWAS statistical noise and to develop a systematic strategy to retrieve these hidden variants. Employing an integrative approach, which combines protein-protein interactions with association data from GWAS for 6 common diseases, we found that associated-genes at less stringent significance levels (*p* < 0.1) with any of these diseases are functionally connected beyond noise expectation. This functional coherence was used to identify disease-relevant subnetworks, which were shown to be enriched in known genes, outperforming the selection of top GWAS genes. As a proof of principle, we applied this approach to breast cancer, supporting well-known breast cancer genes, while pinpointing novel susceptibility genes for experimental validation. This study reinforces the idea that GWAS are under-analyzed and that missing heritability is rather hidden. It extends the use of protein networks to reveal this missing heritability, thus leveraging the large investment in GWAS that produced so far little tangible gain.

## 1. Introduction

Genome-wide association studies (GWAS) hold the promise revealing common variants that are associated with disease risk. These studies have identified numerous genetic risk factors for many common phenotypes, such as diabetes, Crohn’s disease or height (http://www.genome.gov/gwastudies/) [1,2,3,4]. However, the enthusiasm surrounding GWAS for many complex diseases was tempered by the observation that the risk variants identified conferred only a small increment in risk, thus explaining a very small fraction of the genetic variation that we expect to exist, and leaving open the question of what may explain the remaining heritability [5]. Rare variants, epistasis, epigenetics and genotype–environment interactions are possible explanations, but may also just imply that complex traits truly are affected by thousands of variants of small effect.

Because of the large multiple hypothesis correction needed to evaluate thousands of candidate loci individually, traditional single-SNP (single nucleotide polymorphism) GWAS analysis suffer from lack of statistical strength to detect small effect size variants. SNPs are required to attain a very stringent genome-wide significance threshold (<10^−8^) [6,7], and the ones that do not pass it are often ignored, when they may in fact be true associations with effects that are too small to be individually detected. Indeed, a recent study demonstrated that a total of 45% of the height variance could potentially be explained by ~300,000 SNPs without regard to the significance of their association, a nearly tenfold increase relative to the 5% explained by published and validated individual SNPs [8]. The International Schizophrenia Consortium also found that a collection of thousands of nominally significant SNPs collectively capture over one-third of the heritability for schizophrenia, a disease that has proven particularly refractory to the discovery of large effect alleles [9]. These results suggest that SNPs with individual small effects could collectively add a substantial genetic contribution, although they will remain individually difficult to detect through GWAS and, hence, hidden within the statistical “noise”. As a result, a large fraction of the genetic information which may emerge from GWAS remains unused and much of the investment is lost.

Inspired by this hypothesis put forward by classical quantitative genetic studies and realizing the limitations of conventional single-marker association, we addressed the missing heritability problem, aiming to demonstrate that there are indeed risk variants within GWAS statistical noise and develop a systematic strategy to retrieve these hidden variants.

Genetic associations are usually challenging to interpret without a biological context. Moreover, each high-throughput technique, such as genotyping or expression microarrays, independently possess high noise, generating the need of integration with biological data from multiple sources, which has the potential to provide functional links to bridge the knowledge gap between the genetic variants and the phenotypes. Integrative analysis of GWAS and expression data with independent biological knowledge under a rational biological hypothesis, e.g., co-expression network [10,11,12,13,14], protein-protein interaction (PPI) network [15,16,17,18,19,20,21,22,23,24], pre-defined gene sets, such as the Kyoto Encyclopedia of Genes and Genomes (KEGG) database or the Gene Ontology (GO) annotations [4,25,26,27,28,29], or co-evolution information [30] has been shown to be effective in the identification of pathways involved in several diseases and discovery of better predictors than individual genes [31].

In particular, protein-protein interaction networks being based on the physical and direct interaction among proteins, represent one of the strongest indications of functional relationship between genes. Interacting proteins were shown to often share similar functions, participate in the same biological process and contribute to related phenotypes [32,33,34]. Moreover, it has been shown that protein products of disease causing genes tend to be closer to each other in a protein-protein interaction network, and therefore interacting partners of previously known disease-associated genes have been used in the prediction and prioritization of new gene candidates [30,35,36].

In the case of GWAS, PPI data was used to identify disease-causing pathways in complex diseases, mining the data for subnetworks maximizing the disease-association [15,16,19,20,22]. These approaches were based on the idea, supported by previous pathway-based approaches, that although hundreds of genes are involved, they are not randomly distributed with respect to their biological function, but often clustered in common molecular pathways [4,27]. Others have used PPI data to look for significant physical connectivity among proteins encoded by genes in loci associated to disease [37,38]. The novelty in our work is to extend its use to reveal disease-relevant genes buried in the statistical noise.

We here show that PPIs can be used to extract disease-relevant genes buried in the GWAS’ statistical noise. We show this for a variety of complex diseases, and illustrate the use of this approach to reveal novel candidate risk genes for breast cancer.

## 2. Results

### 2.1. Functional Connectivity beyond Random Expectation at the Range of Genome-Wide Association Studies (GWAS) Statistical Noise

Genes with small effect sizes not detectable at conventional levels of significance may be accounting for substantial heritability in complex diseases. Thus, less stringent levels of statistical significance should be explored in the analysis of GWAS data. The challenge resides on how to extract relevant biological information within a statistical range where false positives vastly dominate. We anticipate that disease-causing genes, assumed to be involved in similar processes, would be closer to each other in a protein-protein interaction network, interacting with proteins implicated in the same phenotype more frequently than expected by chance. Hence, mapping of GWAS-associated genes into protein-protein interaction data may reveal functionally coherent networks, which we hypothesize distinguish potentially relevant genes from false positive hits.

We first tested whether such functional coherence at less stringent statistical levels is observed for a set of different diseases, breast cancer, neuroblastoma, type 1 diabetes, multiple sclerosis, systemic lupus erythematosus and Parkinson’s disease (Table S1). Proteins unrelated to the disease are expected to be randomly distributed on the PPI network, while disease-relevant proteins are expected to more often establish direct interactions between themselves and be more rarely found isolated in the network. So, we have calculated the percentage of direct interactions and isolated nodes for sets of proteins encoded by genes selected at different *p*-value cutoffs (0.5 < −Log_10_*p* < 1.5), and compared with what would be expected from statistical noise, which was simulated using 1000, equal sized, random sets of proteins from the network. Gene-wise *p*-values, corrected for gene size and linkage disequilibrium (as described in the Experimental Section), were first calculated, using MAGENTA (Meta-analysis gene-set enrichment of variaNT associations), from the SNP association results for each disorder, taking into account SNPs that mapped within an extended boundary of 10 kb from each gene. Then, genes selected according to different gene-wise *p*-values thresholds were mapped to the corresponding protein in a human protein-protein interaction network.

Sets of disease-associated proteins were found to establish significantly more direct interactions than the random sets (0.001 < *p* < 0.041) for breast cancer, neuroblastoma, systemic lupus erythematosus (SLE) and type 1 diabetes (T1D), when a cutoff of −Log_10_*p* < 1.5 was used (Figure S1A). The significance is maintained at lower *p*-values cutoffs in the case of T1D and neuroblastoma. A significantly higher percentage of direct interactions is observed for −Log_10_*p* < 1, for the remaining GWAS datasets analyzed, Parkinson’s and multiple sclerosis (MS).

The percentage of isolated nodes in the network among disease-associated proteins was significantly smaller than in random sets (0.001 < *p* < 0.048), for – Log_10_*p* cutoffs between 0.5 and 2 in all GWAS datasets, with the exception of MS (Figure S2A).

**Figure 1:**
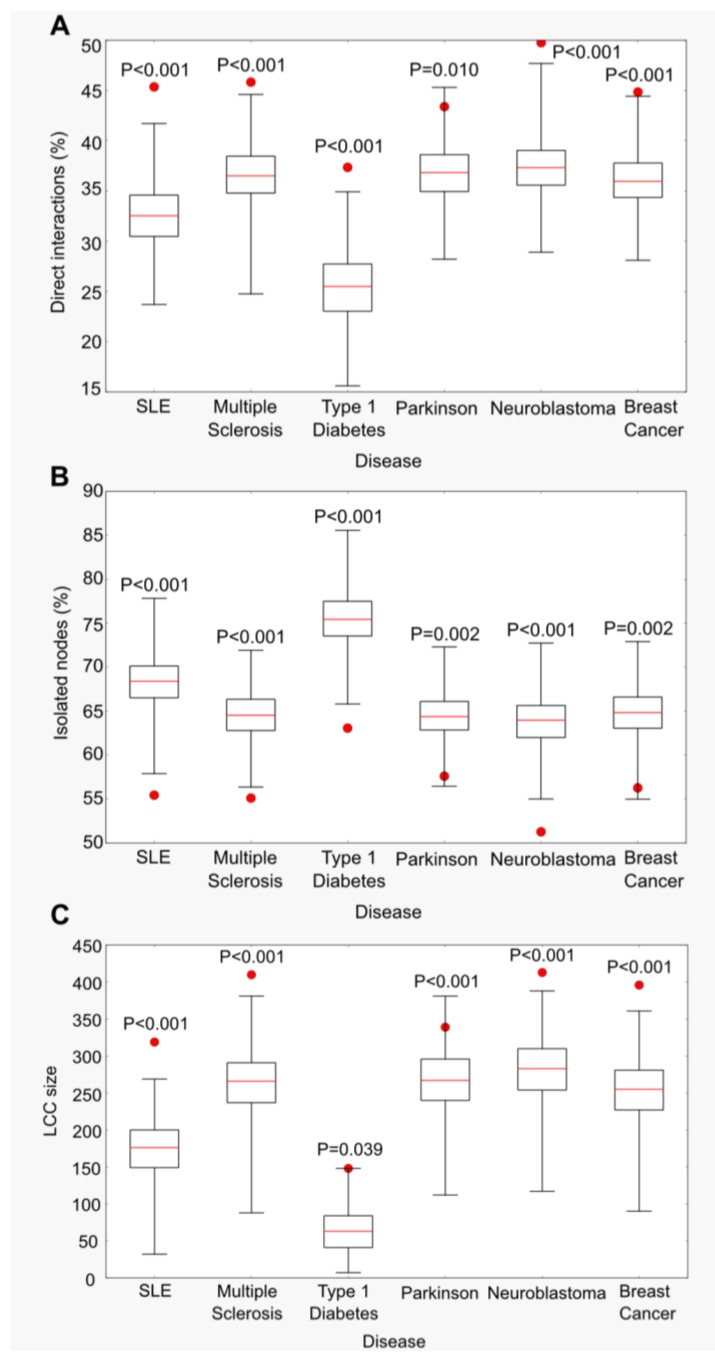
Proteins encoded by genes selected at –Log_10_ gene-wise *p*-values <1 are functionally related in a protein-protein interaction (PPI). Red circles represent the real value obtained for each genome-wide association studies (GWAS) dataset analyzed. Box plots represent the percentage of direct interactions (**A**) and isolated nodes (**B**) and the largest connected component (LCC) size (**C**) in the 1000 random samples of proteins, by disease. Empirical *p*-values are shown.

Based on these observations, we established −Log_10_*p* = 1 as the cutoff value, the lowest gene-wise *p*-value for which, for all the GWAS datasets analyzed, the percentage of direct interactions between disease-associated proteins was significantly higher (Figure 1A) and the percentage of isolated nodes significantly smaller (Figure 1B) than random expectation. These results are maintained if the analysis was restricted to high confidence interactions only, with the exception of Parkinson’s (Figure S1B and Figure S2B).

Taken together these results suggest that genes encoding functionally connected proteins associated with these diseases reside within GWAS statistical noise, revealing that there is indeed unexplored relevant biology at this statistical level.

### 2.2. Functionally Connected Groups Are Enriched in True Disease Genes

Given our observation that there is functional connectivity in GWAS for a number of complex diseases, and thus potentially relevant biology, at the range of −Log_10_*p* < 1, we next questioned whether this functional coherence can be used to identify disease-relevant subnetworks. If within GWAS statistical noise there are indeed proteins relevant for the disease and not just random positive hits, their functional proximity is expected to be translated in a larger group of interconnected proteins. Following this hypothesis, as a proof of principle approach, we used the concept of largest connected component (LCC), which is the largest set of interconnected proteins of the network likely involved in a small number interrelated biological processes. We compared the size of the LCC generated by disease-associated proteins with the size expected if all these proteins are just noise, which was simulated by 1000 random sets of network proteins. We then used curated lists of known candidate genes (as described in the Experimental Section) to test whether these functionally coherent subnetworks contain true biological insight into the diseases.

Disease-associated proteins were found to be interconnected in a significantly larger LCC, when compared to the same number of random proteins from the network, for −Log_10_*p* cutoffs < 1 in all GWAS datasets (Figure 1C). The size of the largest connected component varied from 148 proteins in type 1 diabetes to 413 proteins in neuroblastoma. LCC size is not correlated with the size of the dataset, but rather with the underlying genetic architecture of the disease. For instance, LCC sizes were smaller for SLE and particularly type 1 diabetes, two immune related diseases with a main genetic contribution of the major histocompatibility complex (MHC) genes. The existence of these strong effect genes may imply a small number of low effect genes, which translates into a smaller LCC.

We next evaluated the biological plausibility of our LCC-based filtering approach, by comparing the performance of the genes selected in the LCC against a list of known candidates, with the one obtained for all genes selected at the same gene *p*-value cutoff. If the incorporation of protein interaction information as a strategy to select disease-relevant proteins from all proteins encoded by disease-associated genes at −Log_10_*p* < 1 is meaningful, an enrichment of known disease genes in the LCC is expected. Furthermore, we wanted to know if the use of these subnetworks provides additional insight into diseases than association data alone, thus the performance of our LCC gene selection approach was also compared with the one achieved by the selection of the same number of GWAS top genes. Candidate gene lists were obtained for breast cancer, multiple sclerosis, type 1 diabetes and Parkinson’s disease from curated databases [39,40,41,42].

**Figure 2:**
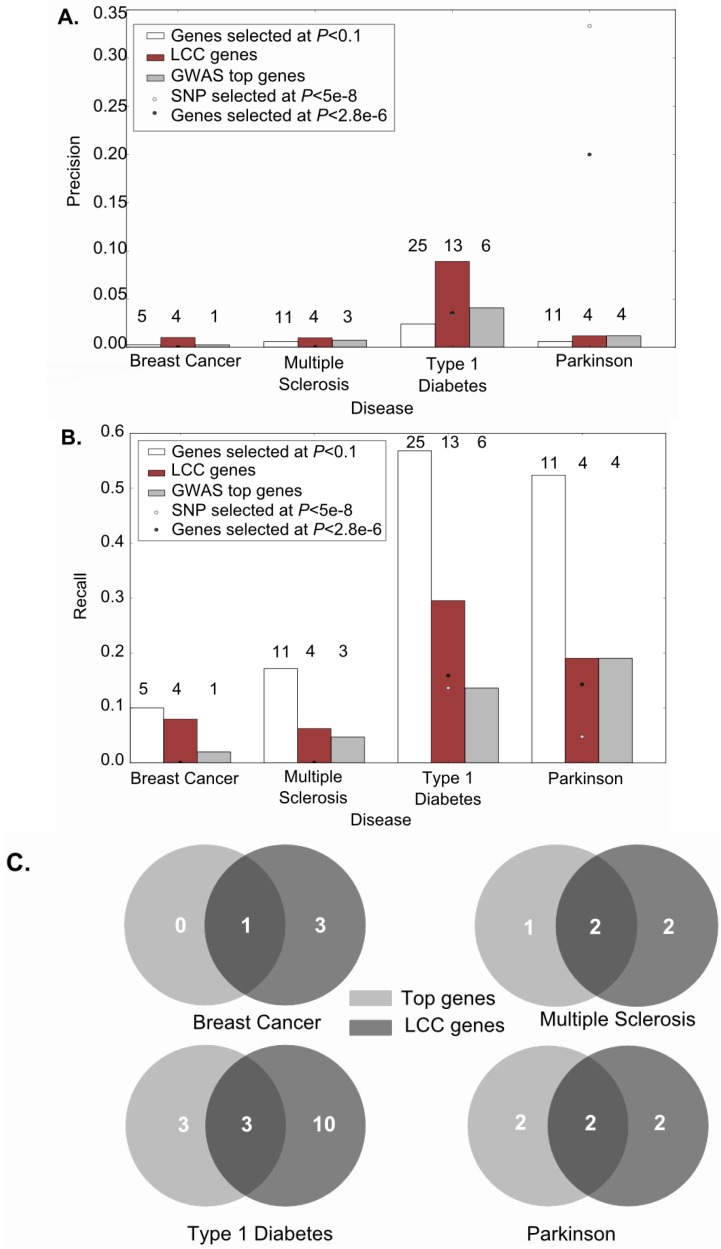
Largest connected components contain true biological insight into diseases. (**A**) Precision, by disease, of five sets of genes against a list of known diseases candidates (*n* = 50, 64, 44 and 21 for breast cancer, multiple sclerosis, Parkinson’s and type 1 diabetes, respectively). The sets of genes evaluated for precision against the lists of known candidates were: the set of genes selected at a gene wise *p*-value cutoff of 0.1 (white bar) (*n* = 1934, 1894, 1035, 1907 in breast cancer, MS, Parkinson’s and type 1 diabetes, respectively), the set of genes included in the LCC obtained from the previous selection (red bar) (*n* = 395, 410, 146 and 337 in breast cancer, MS, Parkinson’s and type 1 diabetes, respectively), the same number of GWAS top genes than the ones included in the LCC (grey bar), the set of genes surviving Bonferroni correction over SNPs or genes (grey and black dots, respectively). Numbers above the bars are the number of known candidates included in each gene selection set; (**B**) Recall, by disease, of the same sets of genes against the lists of known candidates. Numbers above the bars are the number of known candidates included in each gene selection set; and (**C**) Venn diagrams showing, for each disease, the overlap between known candidate genes retrieved by LCC genes (dark grey circle) and by the same number of GWAS top genes (light grey circle).

Figure 2A shows that, with the exception of Parkinson’s disease, the precision achieved by genes included in the LCC was 1.3–4-fold higher compared with the one achieved by the same number of GWAS top genes, suggesting that our selection was more accurate than selecting only the major effect genes. In addition, genes included in the LCC presented a higher or similar precision than all the genes selected at the same statistical level, for all 4 diseases. In other words, genes included in the LCC are 1.6–4-fold enriched for known candidates compared with all selected genes at the same statistical level, demonstrating that our approach based on PPIs to the “GWAS noise” uncover true disease-associated genes.

Remarkably, a mean 2-fold higher proportion of known genes for all diseases (except for Parkinson’s disease) was retrieved by LCC selected genes, compared with the top-gene selection, suggesting that additional relevant low effect genes are being captured (Figure 2B), genes that would be otherwise hidden in the statistical noise. In fact, the overlap between known genes in top-gene and LCC-based selections in Figure 2C shows that LCC captures the majority of known genes present in the GWAS top genes and also additional true disease genes with more modest associations. As expected, genes included in the LCCs had a lower recall compared with all genes selected at the same cutoff, since LCC genes are a subset of this selection (Figure 2B). LCC size was not correlated with the measured precision or recall.

A similar mean fold increase (2-fold) in the precision and recall of LCC genes *vs*. top genes was observed when the high confidence interaction network was used. Concerning all the genes selected at the same statistical level, a mean increase of 3.2-fold in precision was observed for our LCC-based selection (data not shown).

Taken together, these results showed that our selection of functionally connected genes based on the largest connected component is an efficient approach to identify true disease genes.

### 2.3. A Case Study: Breast Cancer Largest Connected Component (LCC) Genes Were Supported by Multiple Sources of Experimental Data

Having demonstrated that the largest connected component contains relevant disease genes, we further explored the performance of this network for the prediction of novel genes, using breast cancer as a case study. We have taken advantage of the large amount of experimental data available for breast cancer to validate our network-based disease gene predictions.

**Figure 3:**
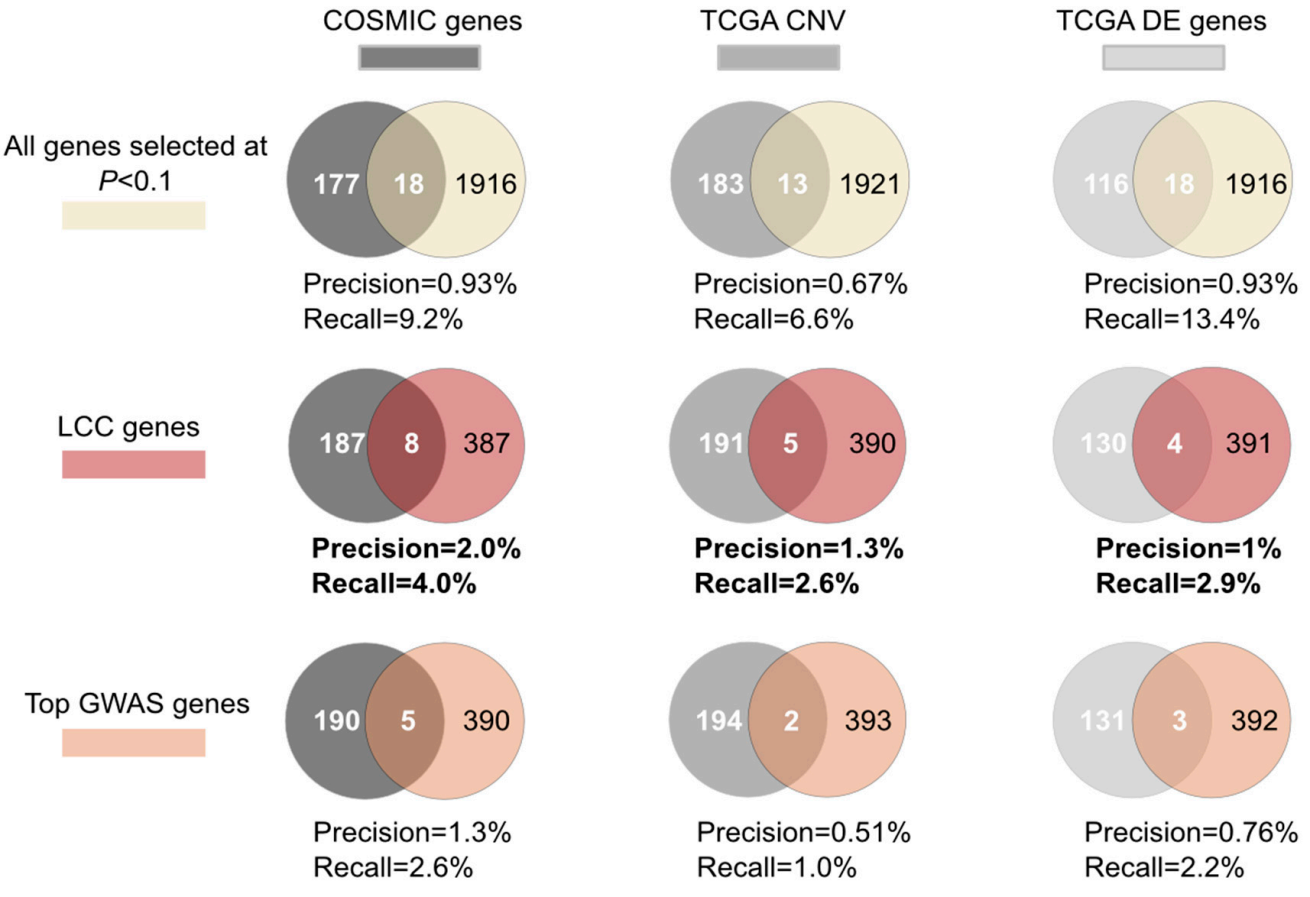
LCC performs better than GWAS top genes in retrieving known breast cancer genes. Venn diagrams showing the overlap between genes reported to be differentially expressed (retrieved from the TCGA data portal, using the default parameters (−0.5 < Log2 < 0.5; frequency = 40%) or to harbor copy number abnormalities (retrieved from the TCGA data portal, using the default parameters −0.5 < Log2 < 0.5; frequency = 20%) or somatic mutation (genes with at least five cases reported in COSMIC database) in breast cancer, with each of the sets of genes selected from the breast cancer GWAS dataset by the previous gene selection approaches (all genes selected at a gene-wise *p*-value <0.1, LCC genes and top GWAS genes, represented in light yellow, red and orange circles, respectively).

The largest connected component generated by genes selected at −Log_10_*p* < 1 from the breast cancer GWAS dataset was composed of 396 proteins. Using the Catalogue of Somatic Mutations in Cancer (COSMIC) [43,44] and The Cancer Genome Atlas (TCGA) data portal (http://tcga-data.nci.nih.gov/tcga) to retrieve genes reported to be differentially expressed or harbor somatic mutations or copy number abnormalities in breast cancer, we examined the potential role in breast cancer of the genes selected by each of the previous gene selection approaches (LCC-based selection, top-GWAS gene selection or all genes selected at *p* < 0.1). The LCC-based selection presents a 1.3- to 2.5-fold increase in the precision and recall compared with top-gene selection (Figure 3). The higher fold increase is observed using the CNV gene list. Overall, the LCC performs better in retrieving genes with somatic mutations, including 8 genes (*HRNR*, *JAK2*, *JAK3*, *MAP3K1*, *NRAS*, *PIK3CA*, *PTEN*, *SOS1*) with mutations reported in more than five patients. Additionally, five reported CNV genes (*EPPK1*, *FGF19*, *GDF6*, *WDYHV1*, *YWHAZ*) and 4 differentially expressed genes (*BMPR1B*, *EEF1A2*, *LTF*, *PGR*) were also present in the LCC.

Gene set enrichment using The database for annotation, visualization and integrated discovery (DAVID) [45,46] revealed that the breast cancer LCC was significantly enriched in FGF, MAPK, Erb, neurotrophin, B-cell and T-cell receptors signaling pathways (6.03 × 10^−5^ < *p* < 0.018), as well as in several KEGG cancer pathways (3.4 × 10^−4^ < *p* < 0.048) (Table S2). Proteins included in the breast cancer LCC were also enriched for generic and breast cancer genes compiled in the genetic association database (GAD). According to the University of Copenhagen Diseases database (http://diseases.jensenlab.org/Search), which collects disease associations derived via automatic text mining of the biomedical literature, 30% of the LCC genes are associated with some type of cancer, compared with only 9% of the top genes. Additionally, among the 396 genes of the breast cancer LCC, 31 have been cataloged as causally implicated in cancer by the Cancer Gene Census [47], compared with 7 among the top genes. To explore this observation and further examine the specificity of the genes in our network, we analyzed the presence of each of these genes in the networks generated from the other five diseases GWAS and derived a score for each gene in order to prioritize the genes for specific association with breast cancer. This analysis revealed that the majority of the genes (~70%) were present only in the breast cancer network, 5.5% of which were present in the breast cancer known gene list. The other cancer dataset analyzed, neuroblastoma, had 10% of LCC genes in common with breast cancer, the majority of which (~65%) are likely generic cancer genes, since they are not present in any other disease network.

### 2.4. Novel Breast Cancer Susceptibility Genes

**Figure 4:**
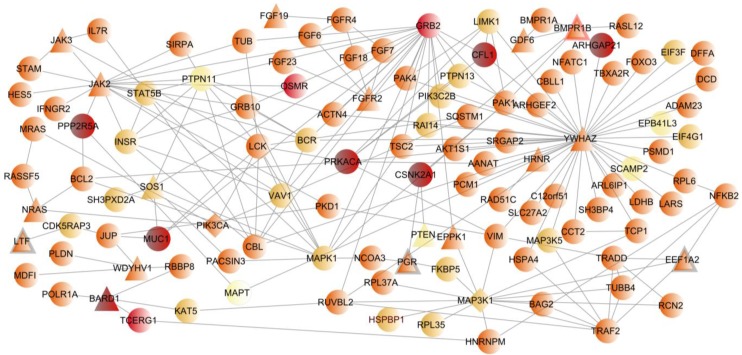
Breast cancer network. This network illustrates the 19 known breast cancer genes included in the breast cancer LCC and their first neighbors. Nodes are colored based on a score reflecting their presence in an additional LCC cancer dataset (neuroblastoma) and in the LCCs for the four unrelated diseases. A darker color represents a higher score, which means a higher specificity for cancer. The shape of the node reflects the presence of each gene in breast cancer gene lists (genes associated with breast cancer in NextBio, genes with somatic mutations, copy number abnormalities or differential expression obtained from COSMIC database and TCGA data portal). Circular nodes are proteins absent from the four lists, triangular nodes are proteins present in one and diamond nodes in two. A thicker border indicates that the gene was reported to be differentially expressed in breast cancer.

From the 19 LCC known breast cancer genes we built a network with 116 proteins by including their first neighbors present in the LCC network (Figure 4). Among the 116 genes, most have nominally significant *p-*values (*p* < 0.05). The most significant genes included are *FGFR2*, the top finding in the original GWAS publication*,* and *POLR1A* genes *(p-*value *<* 10^−4^*).* The network is mainly centered in five genes, *JAK2*, *MAP3K1*, *YWHAZ*, *GRB2* and *MAPK1.* From these 116 genes in Figure 4, we highlighted those 8 genes that achieved the highest score (present in the LCCs derived from both cancer datasets and in none of the other datasets analyzed) as the best candidates for harboring variants associated with cancer risk. We found encouraging support for already known *loci*, such as *BARD1* and *MUC1* genes. For the majority of the remaining genes, although there are no reported studies on breast cancer, associations with carcinogenesis and other types of cancer have been described [48,49,50,51], so they represent promising novel candidate genes for breast cancer risk.

## 3. Discussion

Recent evidence from classical quantitative genetic analysis suggested that most of the “missing” heritability in complex disorders is likely hidden below the threshold for genome-wide significant associations [8]. How these hidden variants can be identified from a statistical range where false positives vastly dominate remains to be determined.

To demonstrate that there are indeed relevant disease variants within the commonly considered “statistical noise” and to leverage the power of GWAS to uncover these small effect risk variants, we have used an integrative approach combining system-level data from protein-protein interactions with association data from publicly available GWAS for 6 common diseases. Disease-causing genes are likely functionally related [4,32,33,34], converging in similar biological processes. On the other hand, protein-protein interactions (PPI) are one of the strongest indications of a functional relationship between genes. Thus, mapping of GWAS-associated genes into protein-protein interaction data may reveal functionally coherent networks, which we hypothesized distinguish potentially relevant genes from false positive hits.

The first challenge that we addressed here was the selection of a higher than conventional association *p*-value threshold for which we could still identify potentially relevant *loci* within the statistical noise. A threshold that is too low will result in an insufficient number of genes to create a meaningful network, while using high *p-*values will introduce many false positives. Statistical noise is expected to have random connections in the network, whereas relevant disease genes are more likely to establish interactions among themselves [32,33,34,35,36]. Thus functional coherence between proteins, inferred from their proximity in the network, could be used to establish this threshold. Using the percentage of direct interactions and isolated nodes as proxies for network proximity, we showed that genes selected at a gene-wise *p*-value bellow 0.1 were functionally connected beyond noise expectation, simulated by random sets of network proteins. Furthermore, at this *p*-value we showed that genes are connected in a significantly larger LCC than expected by chance. These subnetworks defined by the LCC showed a higher precision and recall in detecting previously known disease candidates, than the selection of the same number of proteins encoded by top-GWAS associated genes, suggesting that this is an efficient approach to capture true disease genes. Our results thus strongly support the hypothesis that there are many relevant susceptibility loci with *p*-value <0.1 hidden in GWAS. A similar conclusion was drawn by the International Schizophrenia GWAS consortium, using an allele score approach, which found that optimal discrimination between cases and controls was achieved only after the inclusion of over 70,000 markers with *p*-values as high as 0.2 [9].

Our approach not only supports the idea from previous studies on classical quantitative genetics that many true variants are still hidden within statistical noise, but further showed that functional coherence inferred from protein networks can be used as a biologically meaningful filter to identify these variants. Recently, network-based algorithms were applied to GWAS data, based on adaptations of the original heuristic search algorithm published by Ideker *et al*. [52] for expression data analysis [16,19,20] or on the Google’s PageRank algorithm [15,22]. Some of these approaches set an arbitrary *p*-value threshold or use genes known to be involved in a particular pathology, termed seeds, which can rarely be found with certainty for complex diseases [16,22,53]. There are, however, other methods that can include all GWAS signals without a priori assumptions of association thresholds [15,19,20]. These approaches are focused on the identification of disease-causing pathways, searching for subnetworks that maximize the association with the disease. On the other hand, our study extends the use of networks to reveal disease-relevant genes buried in the statistical noise, making use of functional relatedness, inferred from proximity in a protein network, to establish a meaningful threshold and select genes that maximize disease biological relevance rather than association. Though network-based association analysis has been explored, not much attention has been paid to the formal validation of the methods and their advantage over single-locus association methods. Only two of the above mentioned studies demonstrated the performance of their methods, by benchmarking their predictions with or without the incorporation of network information against susceptibility genes previously found in association studies. We evaluated the performance of our approach by precision-recall analysis using candidate genes compiled from other layers of biological evidence in addition to association studies, and compared it to scenarios in which only association signals were used for gene prioritization, showing a superior performance of PPI-based gene selection over top-associated gene selection. The low precision and recall absolute values are expected given the incompleteness and noise in the available knowledge in the field of complex diseases genetics, in which several candidate genes have been put forward but few proven to be causally implicated. Nevertheless, the mean sensitivity (recall) estimated with our approach (15.7%) is similar to those obtained for available network-topology based prioritization methods [53].

Available data on protein-protein interactions are inherently incomplete (false negatives) and noisy (false positives). Our analyses were performed in parallel in a PPI network that includes high throughput and small scale interaction data (Global network) and in a high confidence network that has only small scale interactions (High confidence network). The first is less biased but includes a higher false positive rate, whereas the second, based on small scale data alone, has less false positives but is highly biased to the most studied genes, processes and pathways. Results obtained were similar regardless of the different confidence levels of the interaction network.

Besides the above well known general limitation of network-based approaches the results of these methods are highly dependent on specific problems raised by GWAS data, since we were taking a gene-centric approach, such as the combination of evidence of association over multiple SNPs within a gene or the distance threshold to assign SNPs to nearby genes, which are still debatable [25,26,54,55,56]. In this study, we have explored the efficiency of our approach using different methods for gene-wise *p*-value calculation, examining potential biases. Based on a compromise between overcorrection, due to independence assumption, and the minimization of biases, we decided to use a recently developed regression-based method that corrects the most significant values for several confounding effects, including linkage disequilibrium (LD) and gene size [57]. It is important to mention that pathway-based GWAS analysis using the popular minimum *p*-value method should be interpreted with caution due to the highly biased results towards large genes, particularly in the case of neurological disorders given the higher mean size of nervous system genes [58]. Another potential limitation of network approaches is that only genic SNPs are examined, and therefore a critical issue is the distance threshold to assign SNPs to nearby genes. Here we have used a conservative distance threshold (10 kb), since we were not focusing on genetic regulatory relationships, which resulted in a coverage of about 55% of the SNPs meeting the quality control criteria. Given that LD patterns are highly variable in different regions of the genome, a more appropriate definition will probably require a whole genome analysis of LD that adjusts the distance of assignment to the extent of the LD observed for each gene.

Overall, although network-based approaches may have an enormous potential to boost association results, there are also many challenges ahead and space for improvement. For instance, comprehensive analyses of functional elements in the human genome, such as the study recently released by the encyclopedia of DNA elements (ENCODE) project [59], will contribute to more refined SNP to gene mapping schemes and the generalization of these approaches to regulatory networks. Because of the large amount of experimental data available for breast cancer we chose this disease as a case study. The application of our approach revealed cancer-related pathways and genes supported by the experimental data available, namely genes with reported mutations, copy number alterations or differential expression in breast cancer. We have found that the breast cancer LCC network was significantly enriched in several cancer related pathways such as FGF, MAPK, Erb, neurotrophin, B-cell and T-cell receptors signaling pathways. As an additional filter to this LCC network, we built a network with 116 proteins, including the 19 known breast cancer genes present in the LCC network and their first neighbors. By ranking these genes based on their presence/absence in the LCC generated from other cancer GWAS and four additional unrelated diseases, we highlighted 8 genes that achieved the highest score (*i.e*., they are present in both cancer LCCs but not in the four unrelated LCCs) as the best candidates for harboring variants associated with cancer risk. We believe that these candidates are enriched in true positive results with a higher chance of experimental validation, but would have been overlooked if we had only considered individual gene associations, given their modest associations with breast cancer. This list supports previously well-studied breast cancer genes, such as the BRCA1 (breast cancer breast cancer 1, early onset) associated RING (really interesting new gene) domain 1 gene (*BARD1*) [60,61], the mucin 1 (*MUC1*) [62,63] or cofilin-1 genes (*CFL1*) [64,65], but also suggest novel candidates that warrant further investigation in breast cancer. Some of these novel genes, such as *CSNK2A1*, *ARHGAP21* and *PRKACA* have already been somehow implicated (genetic association, differential expression and mutation studies) in others types of cancers, namely neck squamous carcinoma [48], colorectal cancer risk [50], lung squamous cell carcinoma [51] and pituitary tumors [49]; while others, such as *PPP2R5A*, have not been studied in cancer, but may represent good biological candidates, given the involvement of other members of the known tumor suppressor phosphatase 2A family in ovarian, uterine and breast cancer risk [66,67].

## 4. Experimental Section

### 4.1. GWAS Datasets

Summary SNP association results were obtained from the database of Genotype and Phenotype (dbGAP) repository for 6 case-control GWAS in breast cancer, neuroblastoma, systemic lupus erythematosus (SLE), type 1 diabetes (T1D), multiple sclerosis and Parkinson’s disease [68,69,70,71,72,73]. All subjects in these studies were Caucasian of European ancestry and were genotyped on the Illumina HumanHap550 platform (Table S1).

### 4.2. Integration of Gene Association Data with Protein-Protein Interaction Data

Genotyped SNPs were assigned to specific genes if they were located within the gene or up to 10 kb from the gene, using the GRCh37/hg19 genome build. A gene score for each gene was calculated using MAGENTA (Meta-analysis Gene-set Enrichment of variant associations) that can be used to determine gene association *p*-values in the absence of individual-level genotype data [57]. Each gene was assigned the most significant *p*-value among the association *p*-values of all individual SNPs mapped to that gene. A step-wise multivariate linear regression analysis was then used to regress out of this *p*-value the confounding effects of gene size, number of SNPs per kilobase (kb), number of independent SNPs, number of recombination hotspots and the number of linkage disequilibrium units per kb.

Genes selected at different gene-wise *p*-value cutoffs (0.5 < −Log_10_*p* < 5) were superimposed onto their corresponding protein in a large human protein-protein interaction network, converting Entrez gene IDs to Uniprot IDs (release 2010_04). This global PPI network, covering 12372 proteins and 58365 interactions, was built integrating data from six public PPI databases: the Biomolecular Interaction Network Database (BIND), the Biological General Repository for Interaction Datasets (BioGRID), Human Protein Reference Database (HPRD), IntAct Molecular Interaction Database, Molecular Interactions Database (MINT) and the MIPS Mammalian Protein-Protein Interaction (MPPI) [74,75,76,77,78,79,80,81]. A high confidence PPI network was built removing all the interactions detected only by one high throughput technique to control for the quality of the network.

### 4.3. PPI Network Analysis

Functional coherence of proteins encoded by genes selected at different gene-wise *p*-value thresholds was inferred from three network metrics, and compared with those determined for 1000 equal size sets of randomly selected proteins from the human PPI network without any network feature constraints since noise is assumed to be unstructured, random and free from study design bias. An empirical *p*-value was estimated as the fraction of random samples where the value of the network metric assessed is greater (or smaller, depending on the metric assessed) than the observed one. The network properties evaluated were the percentage of direct interactions, determined from the nearest neighbor shortest path length, which is the smallest distance (number of edges) among the shortest paths connecting a given selected protein to all other selected proteins (in other words, the percent of interactions involving two genes in the list of selected proteins); the percentage of isolated nodes, which represent the fraction of selected proteins with no interactions with any other selected protein; and the size of the largest connected component (LCC), the largest group of selected proteins that are all reachable from each other in the network. All analyses were performed both on the high confidence and on the global PPI networks.

All network calculations were performed using python module Network X and networks were visualized in Cytoscape [82].

### 4.4. Performance against Benchmarks

To evaluate the performance of the genes included in the LCC in retrieving known candidate genes for a disease, the precision and recall against curated lists of disease candidate genes were calculated. Precision (True positives (TP)/(TP + False positives (FP))) is the proportion of known candidate genes among the selected genes, while recall (TP/(TP + False negatives (FN))) is the proportion of known candidate genes retrieved by the selection. The precision and recall calculated for the genes included in the LCC were compared to those determined using two other gene selection criteria: (a) all genes selected at the same gene *p*-value cutoff used to derive LCC; and (b) the same number of top scoring genes (ranked according gene-wise *p*-values) as those included in the LCC.

Curated lists of candidate genes were obtained for type 1 diabetes (T1D), multiple sclerosis (MS), Parkinson’s disease (PD) and breast cancer. For T1D, 56 susceptibility genes identified by GWAS were retrieved from T1Dbase (http://www.t1dbase.org) [39], 69 MS candidate genes were retrieved from Msgene (http://www.msgene.org) [41], 21 PD candidate genes were retrieved from PDgene (http://www.pdgene.org) [42], and 50 breast cancer genes were obtained using NextBio analysis tool (Cupertino, CA, USA), a curated and correlated repository of experimental data derived from an extensive set of public resources (e.g., ArrayExpress and GEO) [40].

### 4.5. Case Study: Breast Cancer

In order to rank proteins included in the breast cancer LCC by cancer specificity and reproducibility, a prioritization system was created, assigning a score to each protein based on their presence in the LCCs derived from each of five other disease datasets. Each protein included in the breast cancer LCC had an initial arbitrary score of 0.5. Depending if we were selecting for breast cancer specific genes or cancer generic genes, neuroblastoma was used as a replication dataset. In the case of generic cancer genes prioritization, a value of 0.5 was added to the initial protein score if the protein was present in the neuroblastoma LCC and one fourth of 0.5 was subtracted for each other disease dataset LCC where the protein was present. For breast cancer specific genes, neuroblastoma was treated as any other disease and one fifth of 0.5 was subtracted from the score for each LCC in which the protein was present.

Additional lists of breast cancer genes were used to further validate our gene-selection approach: genes with somatic mutations reported in breast cancer available in COSMIC database [43,44]; genes with copy number abnormalities and genes differentially expressed in breast invasive carcinoma selected according the default options (−0.5 < Log2 < 0.5; frequency = 40% and 20% for expression and copy number, respectively) from The Cancer Genome Atlas (TCGA) Data Portal (http://tcga-data.nci.nih.gov/tcga).

## 5. Conclusions

In conclusion, our results have demonstrated a common principle to GWAS data, using an integrative analysis of GWAS for a number of complex diseases with system level data from protein-protein interaction: There are functionally connected genes beyond random expectation within the range of GWAS statistical noise, which contain relevant disease biology, outperforming the selection of top GWAS genes. This general observation reinforces the idea that GWAS are under-analyzed and demonstrates, using a different approach, that many true variants are still hidden within statistical noise, highlighting the potential for the development of more sophisticated network-based methods as a means to leverage the large investments in these studies. The application of our approach to breast cancer identified a group of functionally connected proteins with a higher precision/recall in retrieving genes reported to be differentially expressed or harbor mutation or copy number abnormalities in breast cancer. While providing further evidence for well-known breast cancer genes, our analysis also highlighted novel susceptibility genes that warrant further experimental validation.

## Supplementary Materials

Supplementary materials can be found at http://www.mdpi.com/1422-0067/15/10/17601/s1.

## Supplementary Files

Supplementary File 1
