# Supplementary material for: Hope for GWAS: Relevant Risk Genes Uncovered from GWAS Statistical Noise
Source: Int J Mol Sci. 2014 Sep 29;15(10):17601–21. doi: 10.3390/ijms151017601 (PMC4227180; doi:10.3390/ijms151017601)

Supplementary Information

Table S1. GWAS datasets used in the analysis.

Disease	# Cases	# Controls	# SNPs Analyzed [1]	Number of Proteins Used for Each *p*-Value Threshold [2]	GWAS Study	dbGAP Dataset
Multiple Sclerosis	978	883	245 694	2903; 1040; 438; 205; 106; 54; 27; 17; 11; 8	Baranzini *et al.*, 2009	pha002861
Systemic Lupus Erythematosus	1311	1783	237 603	2640; 857; 340; 180; 116; 90; 73; 62; 51; 48	Hom *et al.*, 2008	pha002848
Type 1 diabetes	4000	3999	242 898	2170; 582; 264; 189; 163; 140; 134; 129; 126; 120	Barrett *et al.*, 2009	pha002862
Breast Cancer	1145	1142	225 246	2862; 1015; 386; 150; 75; 31; 15; 9; 6; 2	Hunter *et al.*, 2007	pha002853
Neuroblastoma	1032	2043	223 442	2878; 1071; 437; 191; 105; 53; 27; 13; 7; 4	Maris *et al.*, 2008	pha002845
Parkinson's Disease	1713	3978	221 465	2875; 1047; 415; 182; 93; 43; 28; 16; 12; 10	Simon-Sanchez *et al.*, 2009	pha002868

[1] Only SNPs mapping within RefSeq gene +/− 10 kb were used; [2] values are presented for −Log10*p* value = 0.5; 1; 1.5; 2; 2.5; 3; 3.5; 4; 4.5; 5.

Table S2. Significantly enriched canonical pathways in the breast cancer LCC.

Term	Count	%	*p*-Value	Benjamini
P00021: FGF signaling pathway	22	5.5556	7.7353×10^{-7}	6.0334×10^{-5}
hsa04012: ErbB signaling pathway	15	3.7879	5.1188×10^{-6}	9.7253×10^{-5}
hsa04662: B cell receptor signaling pathway	14	3.5354	4.8063×10^{-6}	1.0653×10^{-4}
hsa04660: T cell receptor signaling pathway	17	4.2929	3.2812×10^{-6}	1.0909×10^{-4}
hsa04722: Neurotrophin signaling pathway	18	4.5455	4.7793×10^{-6}	1.2712×10^{-4}
hsa04650: Natural killer cell mediated cytotoxicity	19	4.7980	2.9961×10^{-6}	1.3282×10^{-4}
hsa04010: MAPK signaling pathway	29	7.3232	1.0688×10^{-6}	1.4215×10^{-4}
hsa05200: Pathways in cancer	32	8.0808	2.5837×10^{-6}	1.7180×10^{-4}
hsa05220: Chronic myeloid leukemia	13	3.2828	2.6239×10^{-5}	3.4893×10^{-4}
hsa04810: Regulation of actin cytoskeleton	23	5.8081	2.4952×10^{-5}	3.6867×10^{-4}
hsa05215: Prostate cancer	14	3.5354	3.2983×10^{-5}	3.9873×10^{-4}
hsa05213: Endometrial cancer	11	2.7778	2.4175×10^{-5}	4.0183×10^{-4}
hsa05214: Glioma	11	2.7778	1.3435×10^{-4}	0.0015
hsa05223: Non-small cell lung cancer	10	2.5253	1.9906×10^{-4}	0.0020
hsa05218: Melanoma	11	2.7778	3.6997×10^{-4}	0.0033
hsa05210: Colorectal cancer	12	3.0303	3.6244×10^{-4}	0.0034
hsa04360: Axon guidance	15	3.7879	4.3509×10^{-4}	0.0036
hsa04510: Focal adhesion	19	4.7980	7.3750×10^{-4}	0.0054
hsa04310: Wnt signaling pathway	16	4.0404	7.0625×10^{-4}	0.0055
REACT_9470: Signaling by FGFR	7	1.7677	2.1350×10^{-4}	0.0059
REACT_11061: Signalling by NGF	19	4.7980	3.2601×10^{-4}	0.0060
hsa04062: Chemokine signaling pathway	18	4.5455	8.7483×10^{-4}	0.0061
hsa04630: Jak-STAT signaling pathway	16	4.0404	9.2863×10^{-4}	0.0062
REACT_9417: Signaling by EGFR	9	2.2727	6.3074×10^{-4}	0.0086
REACT_498: Signaling by insulin receptor	9	2.2727	1.6422×10^{-4}	0.0090

Table S2. *Cont*

Term	Count	%	*p*-Value	Benjamini
hsa05221: Acute myeloid leukemia	9	2.2727	0.0016	0.0099
hsa04622: RIG-I-like receptor signaling pathway	10	2.5253	0.0016	0.0099
hsa04210: Apoptosis	11	2.7778	0.0019	0.0107
P00047: PDGF signaling pathway	21	5.3030	2.8445×10^{-4}	0.0110
hsa04910: Insulin signaling pathway	14	3.5354	0.0021	0.0117
REACT_16888: Signaling by PDGF	10	2.5253	0.0012	0.0134
hsa04520: Adherens junction	10	2.5253	0.0028	0.0147
hsa04114: Oocyte meiosis	12	3.0303	0.0034	0.0175
P04393: Ras Pathway	13	3.2828	9.9547×10^{-4}	0.0192
P00053: T cell activation	15	3.7879	0.0015	0.0226
P00010: B cell activation	12	3.0303	0.0021	0.0229
P00018: EGF receptor signaling pathway	17	4.2929	9.0451×10^{-4}	0.0233
REACT_578: Apoptosis	14	3.5354	0.0026	0.0237
P00031: Inflammation mediated by chemokine and cytokine signaling pathway	27	6.8182	0.0020	0.0253
hsa05211: Renal cell carcinoma	9	2.2727	0.0054	0.0265
REACT_6900: Signaling in Immune system	23	5.8081	0.0035	0.0273
P00005: Angiogenesis	21	5.3030	0.0038	0.0363
hsa04664: Fc epsilon RI signaling pathway	9	2.2727	0.0104	0.0484

Figure S1. Percentage of direct interactions per gene-wise *p*-value for the six GWAS analyzed. For each −Log10 gene wise *p*-value cutoff in the x-axis the percentage of direct interactions was plotted for proteins encoded by disease-associated genes (red line) and for the mean of 1000 equal sized random samples of proteins (blue line), using the global PPI network (**A**) and the high-confidence PPI network (**B**). Dark grey areas represent the range between the 25th and 75th quartiles and light gray areas indicate the range between the minimum and maximum values of the random data. Empirical *p*-values are indicated.

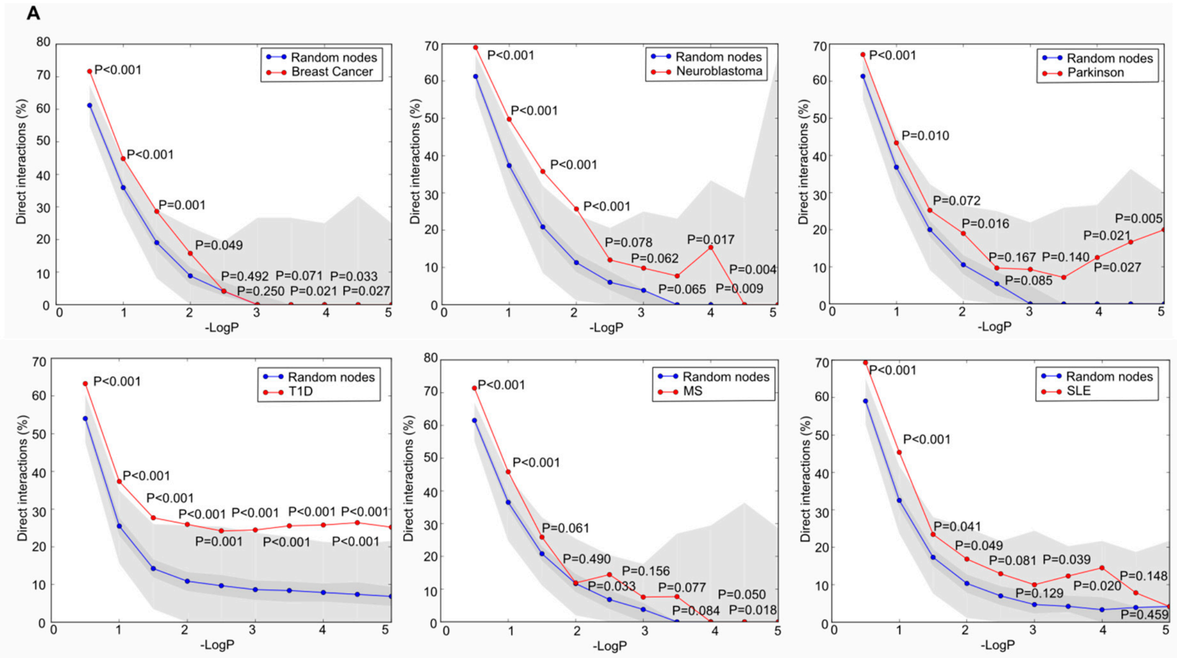

Figure S1. *Cont.*

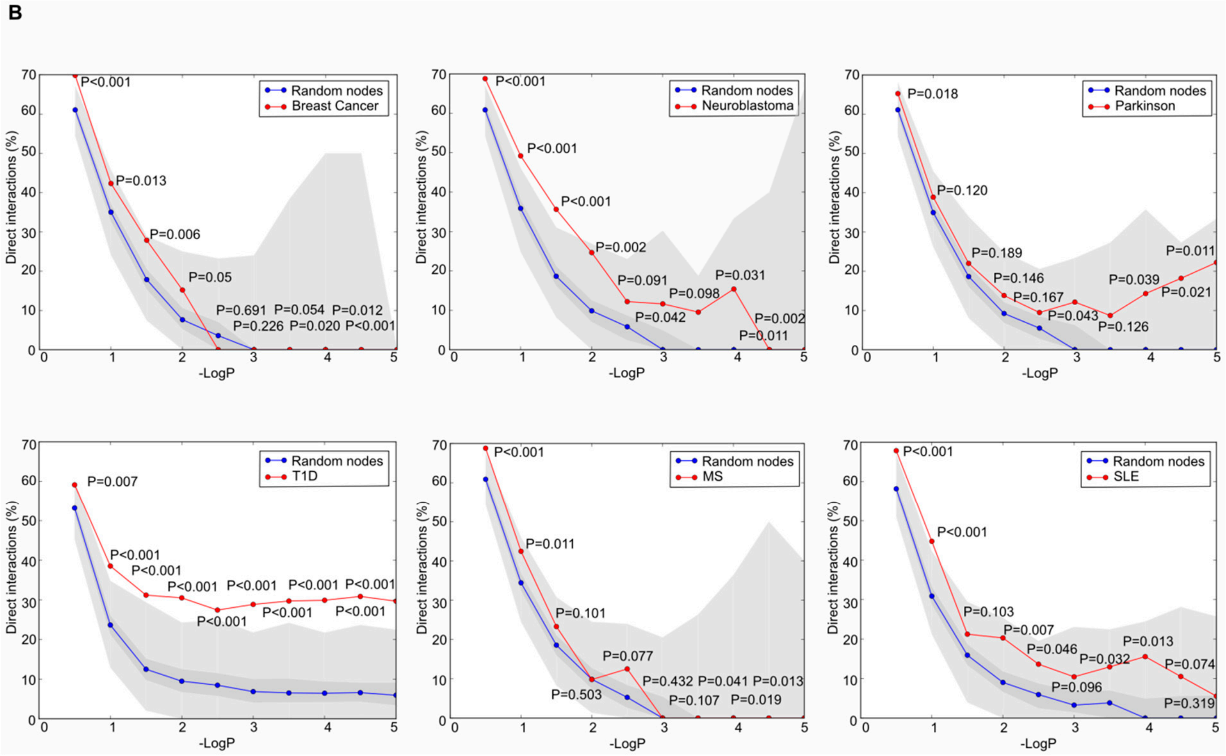

Figure S2. Percentage of isolated nodes per gene-wise *p*-value for the six GWAS analyzed. For each $-\text{Log}_{10}$ gene wise *p*-value cutoff in the x-axis the percentage of isolated nodes was plotted for proteins encoded by disease-associated genes (red line) and for the mean of 1000 equal sized random samples of proteins (blue line), using the global PPI network (**A**) and the high-confidence PPI network (**B**). Dark grey areas represent the range between the 25th and 75th quartiles and light gray areas indicate the range between the minimum and maximum values of the random data. Empirical *p*-values are indicated.

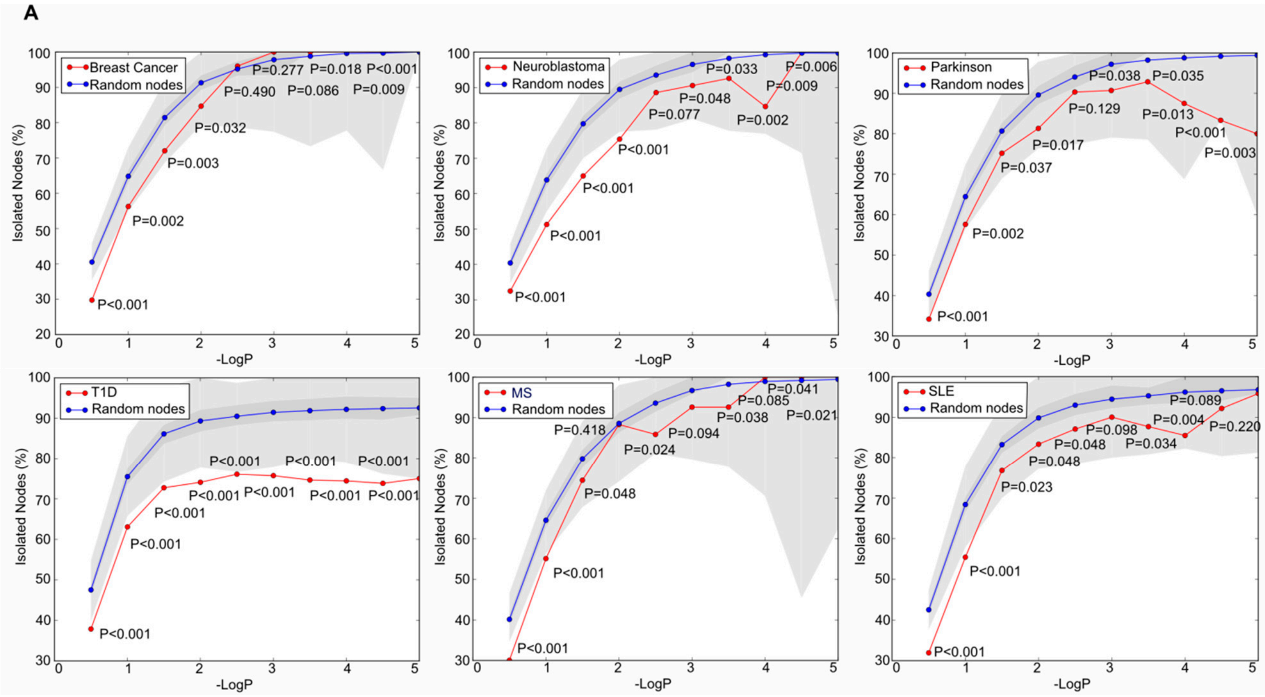

Figure S2. *Cont.*

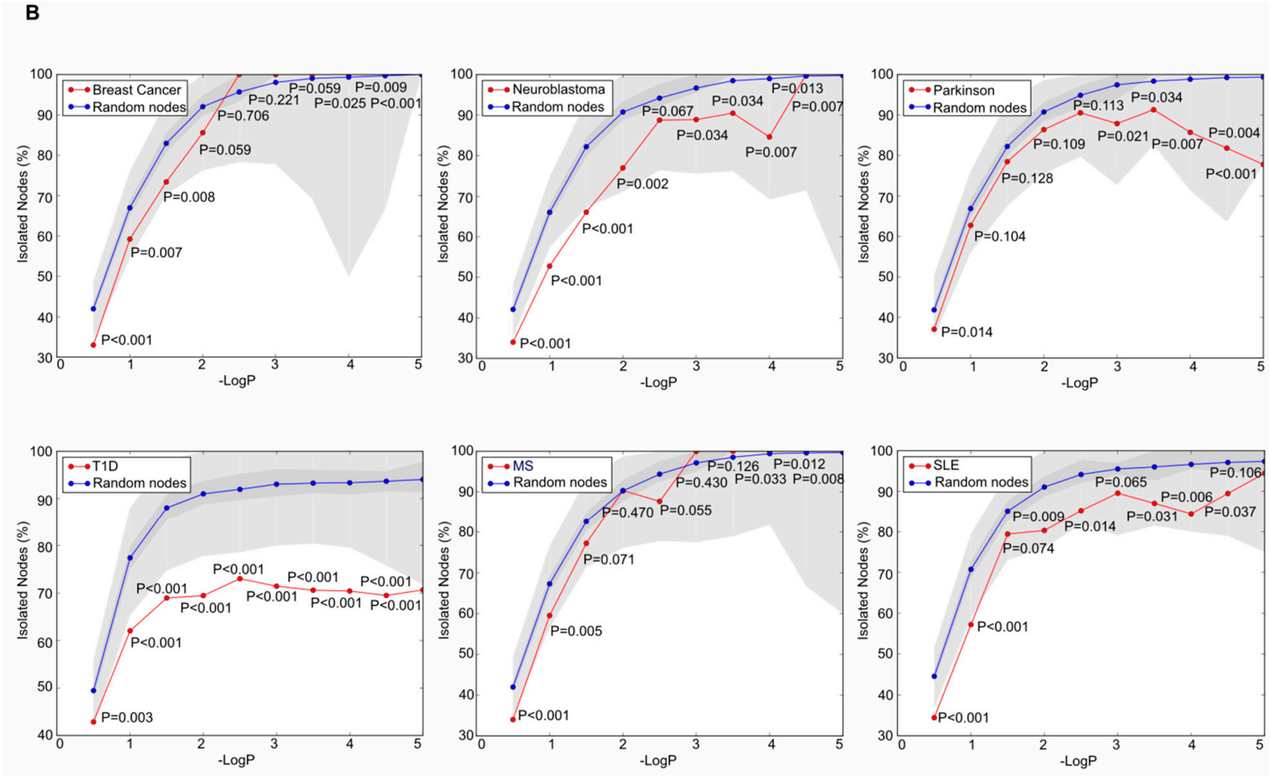

Figure S3. LCC size per gene-wise *p*-value for the six GWAS analyzed. For each $-Log_{10}$ gene wise *p*-value cutoff in the x-axis the logarithm of the LCC size was plotted for proteins encoded by disease-associated genes (red line) and for the mean of 1000 equal sized random samples of proteins (blue line), using the global PPI network (**A**) and the high-confidence PPI network (**B**). Dark grey areas represent the range between the 25th and 75th quartiles and light gray areas indicate the range between the minimum and maximum values of the random data. Empirical *p*-values are indicated.

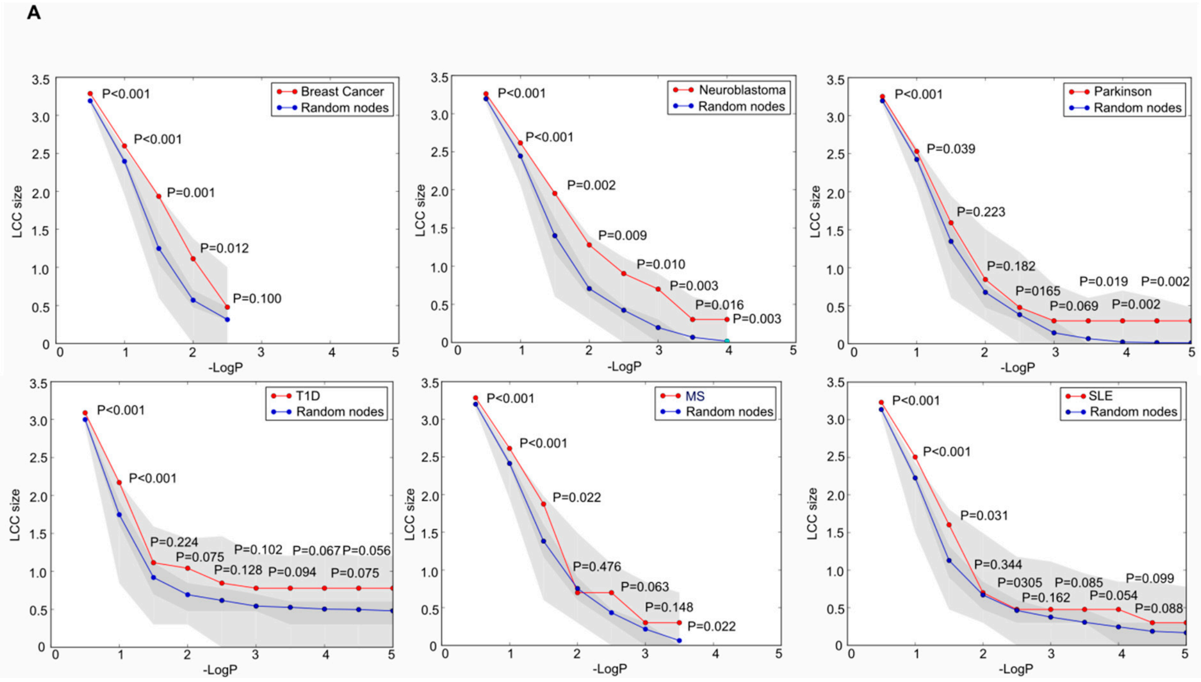

Figure S3. *Cont.*

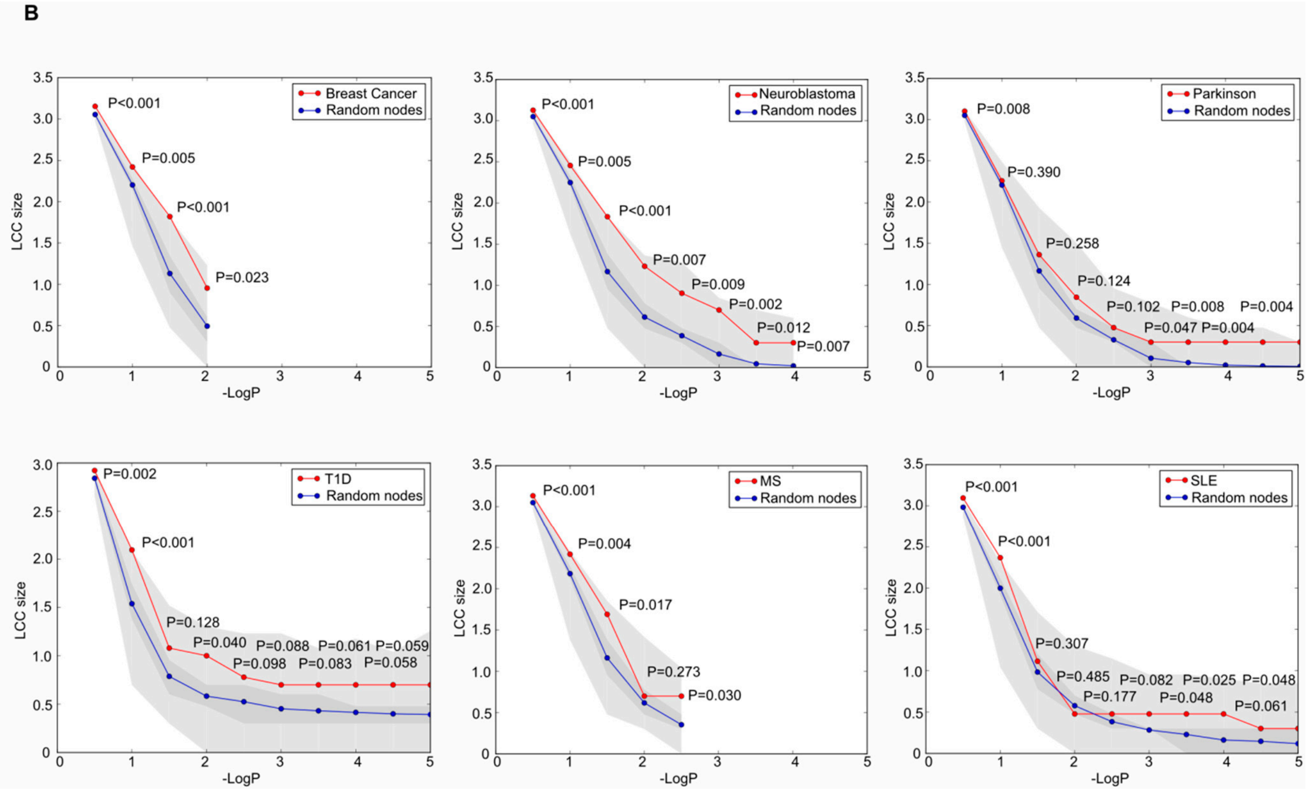

Supplement: Supplementary File 1 [file ijms-15-17601-s001.pdf]
